# Complicated abdominal pregnancy with placenta feeding off sacral plexus and subsequent multiple ectopic pregnancies during a 4-year follow-up: a case report

**DOI:** 10.1186/s13256-016-0808-8

**Published:** 2016-02-11

**Authors:** Chaitali Patel, Joseph Feldman, Chinwe Ogedegbe

**Affiliations:** Hackensack University Medical Center, 30, Prospect Ave, Hackensack, NJ 07601 USA

**Keywords:** Abdominal pregnancy, Ectopic, Laparoscopy, Laparotomy, Peritoneal, Placenta, Recurrent ectopic pregnancy, Sacral plexus, Salpingotomy

## Abstract

**Background:**

Abdominal pregnancy (pregnancy in the peritoneal cavity) is a very rare and serious type of extrauterine gestation that accounts for approximately 1.4 % of all ectopic pregnancies. It also represents one of the few times an ectopic pregnancy can be carried to term. Early strategic diagnosis and management decisions can make a critical difference with regards to severity of morbidity and mortality risk. After an extensive search of the English language medical literature, we are unaware of any case of abdominal pregnancy in which the placenta was receiving its vascular supply from the sacral plexus.

**Case presentation:**

A 26-year-old African-American woman, primigravida, at 16 weeks 4 days’ gestation, presented to our Emergency Department with abdominal pain. She did not complain of any vaginal bleeding. A physical examination revealed mild abdominal tenderness and no blood in the vaginal vault. Laboratory findings corresponded to an increased level of beta human chorionic gonadotropin; magnetic resonance imaging confirmed an abdominal pregnancy. She underwent feticide, administration of methotrexate and a laparotomy was done which was immediately deferred due to perceived increased bleeding risk. She was found to have an intra-abdominal ectopic pregnancy with the placenta attached to her omentum, cul-de-sac and rectosigmoid, with unusual and extensive vascularity from the sacral plexus. A repeat laparotomy was performed 11 weeks later, aimed at removal of the gestational sac and placenta that were left *in situ* on the first laparotomy. This time, we achieved successful removal of the peritoneal gestation, lysis of adhesions, ligation of vascular supply and cautery of the diminished vasculature. Subsequently, she had two ectopic pregnancies, which were managed with both medical and surgical interventions.

**Conclusions:**

Ectopic pregnancies should be identified early and evaluated for the etiology of the presentation. Rarely, an ectopic pregnancy implants at an extratubal location. Today, early intervention saves lives and reduces morbidity, but ectopic pregnancy still accounts for 4 to 10 % of pregnancy-related deaths and leads to a high incidence of ectopic site gestations in future pregnancies. Medical management has emerged as a safe alternative to surgery and holds promise for preservation of future fertility; however, surgery remains an acceptable modality. We found that careful and strategic choice of management pathway can make all the difference to a favorable outcome.

As emergency physicians, we need to be aware of the possibility of abdominal ectopic pregnancy in such presentations and its severe consequences if it remains undiagnosed.

## Background

Ectopic pregnancy occurs when the developing blastocyst becomes implanted at a site other than the endometrium of the uterine cavity. Abdominal pregnancies are rare types of ectopic pregnancies with high rates of maternal morbidity and mortality when encountered anywhere in the world [1]. Abdominal pregnancy accounts for up to 1.4 % of ectopic pregnancies [2]. The prevalence of ectopic pregnancy among women who go to an emergency department with first trimester bleeding, pain, or both ranges from 6 to 16 % [3]. These pregnancies can go undetected until an advanced fetal age and often result in severe hemorrhage [4]. Rates of maternal mortality for ectopic pregnancy in the United States have been reported as high as 20 % [5, 6]. The most common extrauterine location is the fallopian tube, accounting for 98 % of all ectopic gestations, but other possible sites include: cervical, interstitial (also referred to as cornual), hysterotomy scar, intramural, ovarian, or abdominal. Risk factors for ectopic pregnancy should be sought, including prior ectopic pregnancy, current use of an intrauterine device, prior tubal ligation, and *in vitro* fertilization (IVF). The risk of ectopic pregnancy increases with advancing maternal age, age over 35 years being a significant risk factor [1], and one third of the cases are thought to be associated with cigarette smoking [7]. The clinical presentation is variable, and the optimal approach to the evaluation and management of abdominal pregnancy is not well determined [8, 9]. Diagnosis can be made with a transvaginal ultrasound scan (TVS) which can identify an intrauterine pregnancy (IUP) or ectopic pregnancy [10]. In cases where an abdominal ectopic pregnancy is suspected and ultrasound is inconclusive, a diagnostic laparoscopy may be required [11]. Management of these pregnancies has changed dramatically over the years [12]. Medical and surgical interventions to these rare pregnancies are considered based on their presentation. Methotrexate treatment may be administered on a case-by-case basis, but surgical involvement may be imperative for some patients with abdominal pregnancies, particularly those presenting with rupture and who are in a state of hypovolemic shock and compromise. The 1997 to 1999 and 2003 to 2005 *Confidential Enquiries into Maternal Deaths in the United Kingdom* reports highlighted that most of the women who died from ectopic pregnancy were misdiagnosed in the primary care or accident and emergency settings [13, 14].

We present an unusual case that highlights the difficult clinical course of an abdominal ectopic pregnancy managed via strategic procedural intervention, as well as two separate presentations of recurrent ectopic pregnancies over a 4-year follow-up period in the same patient.

## Case presentation

A 26-year-old African-American woman, primigravida, at 16 weeks 4 days’ gestation, presented to our ED with increasing abdominal pain and a positive home pregnancy test. She admitted that she had not been receiving prenatal care. She also asked for a termination of this pregnancy, if we confirmed that it was “abnormal”.

She described 3 out of 10 (mild) bilateral abdominal pain but denied any vaginal bleeding, nausea, vomiting, headache, fever, chills, dysuria or hematuria. She reported 1 to 2 weeks of severe constipation, but denied any melena or hematochezia. She also denied any history of abnormal pap smears, sexually transmitted diseases, or abnormal menses. At the time of her ED presentation she was not on any medications. She had a one pack week cigarette smoking history but denied any alcohol or other drug use. She reported being sexually active with one partner. She further mentioned that she was at a nearby hospital where pregnancy was confirmed and further testing had been done from where she signed out against medical advice (AMA).

Her vital signs were: blood pressure (BP) 125/67 mmHg, pulse 115/minute, temperature 98.7 °F (37.1 °C), respiratory rate 16/minute, body mass index (BMI) 17.75 kg/m^2^, and oxygen saturation (SPO_2_) 99 %.

**Fig. 1:**
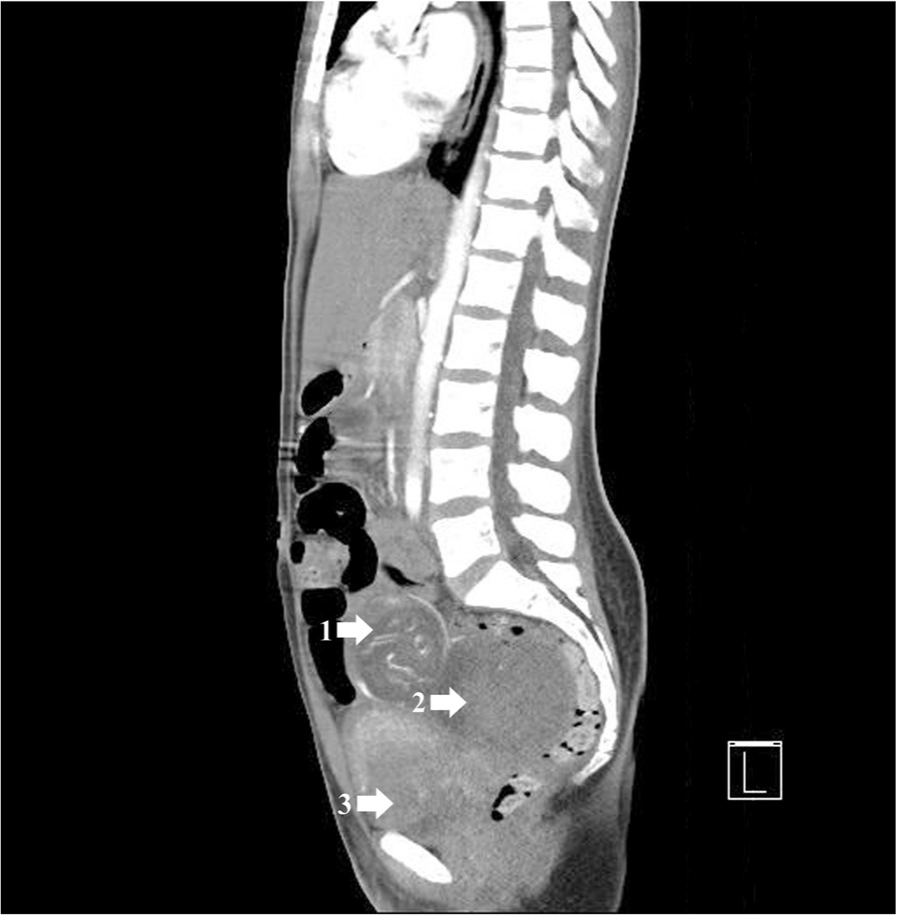
Computed tomography angiogram of abdomen and pelvis. **1** Intra-abdominal pregnancy. **2** Hematoma in cul-de-sac. **3** Uterus

**Fig. 2:**
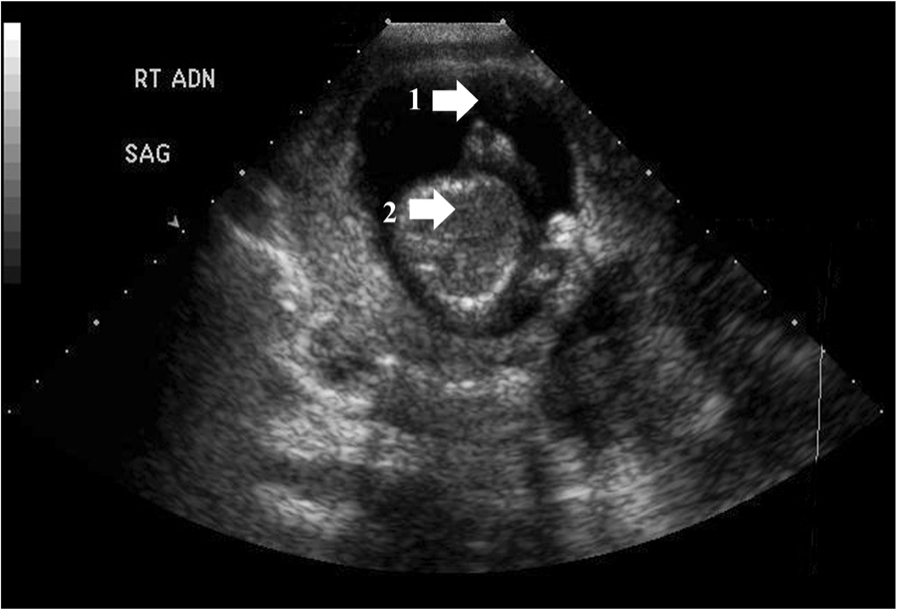
Ultrasound of abdomen and pelvis. **1** Gestational sac. **2** Fetus (extrauterine pregnancy – right adnexa)

Her physical examination was only significant for a soft non-distended abdomen, with mild diffuse lower abdominal tenderness maximal in the left lower quadrant of her abdomen. A pelvic examination, including a speculum examination, documented a normal appearing cervix, no active bleeding, and non-tender adnexa; a bimanual examination confirmed her cervix was closed and her uterus was enlarged, approximately 8 weeks in size. In our ED, she was seen by Obstetrics/Gynecology consult service. Diagnostics included initial and interval laboratory testing, as well as imaging studies, which included an ultrasound of the abdomen (transvaginal and transabdominal), magnetic resonance imaging (MRI) and a computed axial tomography (CAT) scan, (see Fig 1) of her abdomen. The results were as follows:

- Initial laboratory results were hemoglobin (Hb) 9.8 (anemia), hematocrit (HCT) 27.2, platelets 148, white blood cell count (WBC) 7.4, blood urea nitrogen (BUN) 4, creatinine 0.5, electrolytes were within normal ranges, albumin 2.9, partial thromboplastin time (PTT) 33, international normalized ratio (INR) 1.1, prothrombin time (PT) 13.5, beta human chorionic gonadotropin (BHCG) 134,494, fibrinogen 439, human immunodeficiency virus (HIV)-negative and RH positive.
- MRI: intra-abdominal ectopic pregnancy with the placenta connected to the sacral plexus. Ultrasound of the abdomen (transvaginal and transabdominal), (see Fig 2) as well as abdominal CAT scans with and without contrast showed uterus measuring 9.0×5.8×8.2 cm; no IUP; live intra-abdominal pregnancy was present within her pelvis, ventral to the uterus and measuring 7.2×12 cm.

She was counselled on termination of the pregnancy due to the gravity of the medical and surgical comorbidities, and she underwent feticide via ultrasound-guided fetal intracardiac potassium chloride injection on the second day after presentation, gestational age corresponding to 16 weeks and 6 days. She received four alternate doses of methotrexate on the third, fifth and seventh day from presentation with interval leucovorin rescue on alternate days and then had an infraumbilical laparotomy for pelvic exploration and planned fetus removal. A laparotomy was initiated and during the procedure it was found that there was extensive vascularity and attachment of the gestational sac to her omentum, mesentery, loops of bowel, lateral pelvic wall, and predominantly her uterine wall. The vascularity was mainly derived from the sacral plexus and the left external iliac artery, and the patient was deemed inoperable due to the high risk of hemorrhage, need for bowel resection, and potential hysterectomy. Consequently, no attempt was made to remove the gestational sac, the placenta was left *in situ* as well, and the planned fetus removal was deferred to a later date. She was discharged to follow-up at clinic for serial monitoring of her BHCG levels every week, and expectant management. She returned to our ED 11 weeks after feticide with complaints of abdominal pain and vaginal bleeding. A strategic decision was made to bring her to the Operating Room (OR) 2 days later for a second laparotomy, this time with lysis of adhesions, removal of abdominal pregnancy and left salpingo-oophorectomy, which was thought to be the primary site of implantation prior to secondary implantation in her peritoneum. She tolerated the intervention and no adverse or unanticipated event was noted. She was discharged and asked to follow-up with BHCG levels until her levels returned to normal and to return for any further complaints. She adhered to the advice and was counselled not to get pregnant for a year.

Two years later, she presented to our ED with abdominal pain and nausea. Diagnosis was made of a second ectopic pregnancy in her right fallopian tube, with gestational age corresponding to 6 weeks. She received expectant medical management with methotrexate administration: first dose on the day of presentation, second and third doses, 4 and 7 days after the first methotrexate dose was given. Her BHCG levels were monitored and were seen to trend down from 7026 mIU/ml to 5368 and 3739 and complete resolution of this second ectopic pregnancy was noted. She presented with abdominal pain at our ED approximately 8 months after her second ectopic pregnancy. A TVS confirmed a third ectopic pregnancy, at 5 weeks and 5 days of gestation, adjacent to her right ovary. An exploratory laparotomy was performed with right salpingotomy and ectopic removal after she consented to surgery.

## Discussion

Abdominal pregnancy (pregnancy in the peritoneal cavity) is a very rare and serious type of extrauterine gestation that accounts for approximately 1.4 % of all ectopic pregnancies [15]; “it also represents one of the few times an ectopic pregnancy can be carried to term” [16]. Early strategic diagnosis and management decisions can make a critical difference with regards to severity of morbidity and mortality risk.

To the best of our knowledge, our case report represents the first report of an abdominal ectopic pregnancy with a blood supply from the sacral plexus. The consistent clinical presentation of our patient was abdominal pain and nausea, whereas the most common clinical presentation of ectopic pregnancy is early trimester vaginal bleeding and/or abdominal pain [17]. Currently, diagnosis in unruptured ectopic pregnancy is achieved using a combination of transvaginal ultrasonography and measurement of serum BHCG concentrations [11]. Diagnosis can be straightforward when a TVS positively identifies an IUP or ectopic pregnancy [10]. In cases where an ectopic pregnancy is suspected and ultrasound is inconclusive, a diagnostic laparoscopy may be required; this is believed by many to be the ‘gold standard’ investigation in ectopic pregnancy [10]. The patient in our case had all the diagnostic tests, including a laparotomy for the removal of the fetus and the gestational sac during the initial ectopic (abdominal) pregnancy, when she presented with severe abdominal pain. Consequent to the earlier methotrexate administration, repeat abdominal examinations and expectant management, it was thought that the gestational sac, fetus and placenta would continue to be reabsorbed and diminish in volume and complexity, as found at the second laparotomy. The placenta was more easily separated from the sacral plexus and internal iliac vessels with standard surgical procedures, including vascular supply ligation and cautery. “Treatment with pre-operative systemic methotrexate with subsequent laparotomy for removal of the fetus and placenta may minimize potential blood loss, and would be a reasonable approach in the care of a patient with an abdominal pregnancy with placental implantation to the abdominal viscera and blood vessels” [18], as noted here in a 16-week gestation case. During surgery, if the placenta is attached to vital organs it should be left behind [18]. Eleven weeks after the feticide procedure and previous laparotomy, we expected the sac to be completely reabsorbed and eventually not to perform any more invasive procedures. We were surprised at the finding that the products of conception (POC) were not completely reabsorbed and the POC was still at the size of 17 weeks, although it was 11 weeks post-feticide procedure. Reluctance or delay in performing a diagnostic laparoscopy has been highlighted as a factor in fatal cases [19]. The second laparotomy was successful, and on observation of the tubes and uterus, a rupture site was noted on the left tube, which was the reason for the left salpingo-oophorectomy. Post-surgery our patient did well with Hb within normal limits, and no further complaints or complications were noted. A laparotomy should be reserved for patients who present with rupture and are in a state of hypovolemic shock and compromise [11]; however, our patient was deemed unfit for definitive management by laparoscopy because of the extensive vascularity of her placenta from the surrounding structures, and therefore had a laparotomy.

If BHCG concentrations are falling but an ectopic pregnancy has not been excluded, then consideration should be given to performing serial BHCG measurements until levels become undetectable, as rupture can still occur [20]. Laparoscopic procedures are associated with shorter operative times, less intraoperative blood loss, shorter hospital stays and lower analgesia requirements [21–23], compared to laparotomy.

The conclusions and recommendations in the “Practice Bulletin, June 2008, Medical Management of Ectopic Pregnancy” of the American Congress of Obstetricians and Gynecologists (ACOG) state that the results of randomized trials in which systemic methotrexate with tube-sparing laparoscopic surgery were compared showed no difference in overall tubal preservation, tubal patency, repeat ectopic pregnancy, or future pregnancies, based on good and consistent evidence. In contrast to tubal ectopic pregnancies, primary methotrexate therapy for early gestation in abdominal pregnancies has had minimal success [24]. This was different to our case, where the second ectopic (6 weeks) was reabsorbed and our patient’s BHCG levels returned to normal after methotrexate treatment. It further suggests that failure of the BHCG level to decrease by at least 15 % from day 4 to day 7 after methotrexate administration is considered treatment failure requiring therapy with either additional methotrexate administration or surgical intervention.

In our patient, even though her BHCG levels were trending down by 15 % and more with each passing day, a surgical intervention was unavoidable due to the unusual location and unabsorbed gestational sac. The Practice Committee of the American Society for Reproductive Medicine suggested that ovarian and abdominal pregnancies should be diagnosed definitively at the time of surgical exploration, and both conservative surgery and medical therapy may be viewed as appropriate first-line therapies in many early unruptured ectopic pregnancies [25]. As elected in our case, surgical intervention was the initial step in the management of our patient’s ectopic pregnancy.

The strength of our case report was the strategic approach to management, which included a stepwise combination therapy approach, tailored to the patient’s clinical condition, with some input from her and shared decision making with her.

There is a 5 to 20 % risk of a recurrence of ectopic pregnancy with one previous ectopic pregnancy and a risk of 32 % or more following more than one previous ectopic pregnancy [11]. The second ectopic pregnancy in our patient was uneventful with medical management and reabsorption of the POC. With regards to her third ectopic pregnancy, she initially refused any interventions and only accepted methotrexate administration on the day of presentation at 5 weeks and 5 days of gestational age, the second dose on day 3 and the third dose on day 7, after her initial dose, with follow-up of BHCG levels every 3 days. However, she returned 10 days later, with right lower quadrant (RLQ) pain and gestational age corresponding to 7 weeks; laparotomy with planned salpingotomy was identified as the best choice after a shared decision-making process. Our patient, an educated African-American woman had access to our hospital and emergency care, was also employed, but without medical insurance. While talking about her experience, she mentioned her financial constraints and the lack of a proper insurance policy, which had impacted her life and her earlier decisions of signing out AMA. Involving social services and encouraging shared decision making with the patient, regarding her procedure choices, her health and her future reproductive life, were critical in the successful outcome of this case.

## Conclusions

Ectopic pregnancies should be identified early and evaluated for the etiology of the presentation. Rarely, an ectopic pregnancy implants at an extratubal location, such as the cervix, ovary, abdomen, liver, spleen or caesarean section scar [26]. Today, early intervention saves lives and reduces morbidity, but ectopic pregnancy still accounts for 4 to 10 % of pregnancy-related deaths and leads to a high incidence of ectopic site gestations in subsequent pregnancies [27]. Medical management has emerged as a safe alternative to surgery and holds promise for preservation of future fertility [28]. Surgery remains an acceptable, and sometimes necessary, modality for the treatment of ectopic pregnancy [29]. Counselling in these patients, as well as shared decision making, represents an important aspect, as they require a lot of care and understanding of the future risks of similar pregnancies. A careful and strategic choice of management pathway can make all the difference to a favorable outcome.

## Consent

Written informed consent was obtained from the patient for publication of this case report and accompanying images. A copy of the written consent is available for review by the Editor-in-Chief of this journal.

## Abbreviations

AMA: Against medical advice
BHCG: Beta human chorionic gonadotropin
CAT: Computed axial tomography
ED: Emergency Department
Hb: Hemoglobin
IUP: Intrauterine pregnancy
MRI: Magnetic resonance imaging
POC: Products of conception
TVS: Transvaginal ultrasound scan

## References

[1] Farquhar CM. Ectopic pregnancy. Lancet. 2005;366(9485):583–91. 10.1016/S0140-6736(05)67103-6 16099295

[2] Dover RW, Powell MC. Management of a primary abdominal pregnancy. Am J Obstet Gynecol. 1995;172(5):1603–4. 10.1016/0002-9378(95)90504-9 7755078

[3] Murray H, Baakdah H, Bardell T, Tulandi T. Diagnosis and treatment of ectopic pregnancy. CMAJ. 2005;173(8):905–12. 10.1503/cmaj.050222 PMC1247706 16217116

[4] Fisch B, Peled Y, Kaplan B, Zehavi S, Neri A. Abdominal pregnancy following *in vitro* fertilization in a patient with previous bilateral salpingectomy. Obstet Gynecol. 1996;88(4 Pt 2):642–3. 10.1016/0029-7844(96)00213-x 8841236

[5] Onan MA, Turp AB, Saltik A, Akyurek N, Taskiran C, Himmetoglu O. Primary omental pregnancy: case report. Hum Reprod. 2005;20(3):807–9. Epub 2005 Jan 7. 10.1093/humrep/deh683 15640259

[6] Varma R, Mascarenhas L, James D. Successful outcome of advanced abdominal pregnancy with exclusive omental insertion. Ultrasound Obstet Gynecol. 2003;21(2):192–4. 10.1002/uog.25 12601846

[7] Bouyer J, Coste J, Shojaei T, Pouly JL, Fernandez H, Gerbaud L, et al. Risk factors for ectopic pregnancy: a comprehensive analysis based on a large case-control, population-based study in France. Am J Epidemiol. 2003;157(3):185–94. 10.1093/aje/kwf190 12543617

[8] Nkusu Nunyalulendho D, Einterz EM. Advanced abdominal pregnancy: case report and review of 163 cases reported since 1946. Rural Remote Health. 2008;8(4):1087. Epub 2008 Dec. 19053177

[9] Molinaro TA, Barnhart KT. Abdominal pregnancy, cesarean scar pregnancy, and heterotopic pregnancy; UpToDate: http://www.uptodate.com/contents/abdominal-pregnancy-cesarean-scar-pregnancy-and-heterotopic-pregnancy

[10] Condous G, Timmerman D, Goldstein S, Valentin L, Jurkovic D, Bourne T. Pregnancies of unknown location: consensus statement. Ultrasound Obstet Gynecol. 2006;28(2):121–2. 10.1002/uog.2838 16933302

[11] Sivalingam VN, Duncan WC, Kirk E, Shephard LA, Horne AW. Diagnosis and management of ectopic pregnancy. J Fam Plann Reprod Health Care. 2011;37(4):231–40. 10.1136/jfprhc-2011-0073 PMC3213855 21727242

[12] Yao M, Tulandi T. Current status of surgical and nonsurgical management of ectopic pregnancy. Fertil Steril. 1997;67(3):421–33. 10.1016/s0015-0282(97)80064-7 9091325

[13] Lewis G. The Confidential Enquiry into Maternal and Child Health (CEMACH). Saving mothers’ lives: reviewing maternal deaths to make motherhood safe 2003-2005. RCOG Press; London, UK: 2007. Semin Perinatol. 2012;36(1):22–6. doi:10.1053/j.semperi.2011.09.005. https://www.cdph.ca.gov/data/statistics/Documents/MO-CAPAMR-CMACE-2006-08-BJOG-2011.pdf. PMID: 22280861.

[14] O'Herlihy C et al. Deaths in early pregnancy; Centre for Maternal and Child Enquiries (CMACE). Saving mothers’ lives: reviewing maternal deaths to make motherhood safe 2006-2008. London: RCOG Press; 2011. p. 81–4. https://www.cdph.ca.gov/data/statistics/Documents/MO-CAPAMR-CMACE-2006-08-BJOG-2011.pdf.

[15] Karaer O, Ilkgül O, Oruç S. Primary omental pregnancy on the gastrocolic ligament. South Med J. 2007;100(4):403–4. 10.1097/01.smj.0000254211.50103.e6 17458402

[16] Ludwig M, Kaisi M, Bauer O, Diedrich K. Case report: the forgotten child – a case of heterotopic, intra-abdominal and intrauterine pregnancy carried to term. Hum Reprod. 1999;14(5):1372–4. 10.1093/humrep/14.5.1372 10325296

[17] Alkatout I, Honemeyer U, Strauss A, Tinelli A, Malvasi A, Jonat W, et al. Clinical diagnosis and treatment of ectopic pregnancy. Obstet Gynecol Surv. 2013;68(8):571–81. doi:10.1097/OGX.0b013e31829cdbeb. 23921671

[18] Gupta P, Sehgal A, Huria A, Mehra R. Secondary abdominal pregnancy and its associated diagnostic and operative dilemma – three case reports. J Med Case Reports. 2009;3:7382. doi:10.4076/1752-1947-3-7382. PMC2737786 19830195

[19] Robson SJ, O’Shea RT. Undiagnosed ectopic pregnancy: a retrospective analysis of 31 ‘missed’ ectopic pregnancies at a teaching hospital. Aust N Z J Obstet Gynaecol. 1996;36(2):182–5. 10.1111/j.1479-828x.1996.tb03282.x 8798311

[20] Morin L, Van den Hof MC. Ultrasound evaluation of first trimester pregnancy complications; Diagnostic Imaging Committee, Society of Obstetricians and Gynaecologists of Canada. J Obstet Gynaecol Can. 2005;27:581–91. 10.1016/s1701-2163(16)30718-6 16100636

[21] Parker J, Bisits A. Laparoscopic surgical treatment of ectopic pregnancy: salpingectomy or salpingostomy? Aust N Z J Obstet Gynaecol. 1997;37:115–7. 10.1111/j.1479-828x.1997.tb02232.x 9075562

[22] Clausen I. Conservative versus radical surgery for tubal pregnancy. A review. Acta Obstet Gynecol Scand. 1996;75(1):8–12. 10.3109/00016349609033276 8561006

[23] Thornton KL, Diamond MP, DeCherney AH. Linear salpingostomy for ectopic pregnancy. Obstet Gynecol Clin North Am. 1991;18:95–109. 1923258

[24] Zinger M, Rosenfeld D. Failed treatment of abdominal pregnancy with methotrexate. A case report. J Reprod Med. 2001;46(4):392–4. 11354843

[25] Correspondence: A Practice Committee, American Society for Reproductive Medicine, Birmingham, Alabama; Fertility and Sterility® Vol. 100, No. 3, September 2013 0015-0282; https://www.asrm.org/uploadedFiles/ASRM_Content/News_and_Publications/Practice_Guidelines/Committee_Opinions/optimizing_natural_fertility(1).pdf

[26] Walker JJ. Ectopic pregnancy. Clin Obstet Gynecol. 2007;50:89–99. 10.1097/GRF.0b013e31802f4f79 17304026

[27] Marion LL, Meeks GR. Ectopic pregnancy: history, incidence, epidemiology, and risk factors. Clin Obstet Gynecol. 2012;55(2):376–86. doi:10.1097/GRF.0b013e3182516d7b. 22510618

[28] Juneau C, Bates GW. Reproductive outcomes after medical and surgical management of ectopic pregnancy. Clin Obstet Gynecol. 2012;55(2):455–60. doi:10.1097/GRF.0b013e3182510a88. 22510628

[29] Stock L, Milad M. Surgical management of ectopic pregnancy. Clin Obstet Gynecol. 2012;55(2):448–54. doi:10.1097/GRF.0b013e3182510a19. 22510627

